# How Do I Fit through That Gap? Navigation through Apertures in Adults with and without Developmental Coordination Disorder

**DOI:** 10.1371/journal.pone.0124695

**Published:** 2015-04-13

**Authors:** Kate Wilmut, Wenchong Du, Anna L Barnett

**Affiliations:** Perception and Motion Analysis Lab, Oxford Brookes University, Oxford, United Kingdom; McMaster University, CANADA

## Abstract

During everyday life we move around busy environments and encounter a range of obstacles, such as a narrow aperture forcing us to rotate our shoulders in order to pass through. In typically developing individuals the decision to rotate the shoulders is body scaled and this movement adaptation is temporally and spatially tailored to the size of the aperture. This is done effortlessly although it actually involves many complex skills. For individuals with Developmental Coordination Disorder (DCD) moving in a busy environment and negotiating obstacles presents a real challenge which can negatively impact on safety and participation in motor activities in everyday life. However, we have a limited understanding of the nature of the difficulties encountered. Therefore, this current study considered how adults with DCD make action judgements and movement adaptations while navigating apertures. Fifteen adults with DCD and 15 typically developing (TD) controls passed through a series of aperture sizes which were scaled to body size (0.9-2.1 times shoulder width). Spatial and temporal characteristics of movement were collected over the approach phase and while crossing the aperture. The decision to rotate the shoulders was not scaled in the same way for the two groups, with the adults with DCD showing a greater propensity to turn for larger apertures compared to the TD adults when body size alone was accounted for. However, when accounting for degree of lateral trunk movement and variability on the approach, we no longer saw differences between the two groups. In terms of the movement adaptations, the adults with DCD approached an aperture differently when a shoulder rotation was required and then adapted their movement sooner compared to their typical peers. These results point towards an adaptive strategy in adults with DCD which allows them to account for their movement difficulties and avoid collision.

## Introduction

The ability to locomote through the environment without falling or bumping into obstacles is a fundamental skill. One example of such an obstacle is a ‘gap’ or ‘aperture’ that is created by two parked cars, street furniture or a partially blocked doorway. In this situation it is important that the mover is able to make a judgement about the aperture in terms of whether or not a shoulder rotation is needed in order to pass through without collision; throughout this paper this type of judgement is referred to as an ‘action judgement’. Warren & Whang first considered how adults navigate through apertures by asking participants to walk through a range of aperture sizes some of which required a shoulder rotation and others which did not [1]. They found that adults only rotate their shoulders to pass through an aperture when it is less than 1.3 times shoulder width and that this ratio was invariant to body size. They termed this the ‘critical ratio’. These findings extended the geometric scaling model, originally proposed by Warren following work on ‘climbability’ of stair height, which suggests that our ability to judge our own actions is based on our body size [2]. This model proposes that our knowledge of body size allows us to determine whether or not we could step on a given riser height or pass through an aperture without turning laterally. In terms of passing through apertures this allows us to move through the environment efficiently; neither turning every time when it is unnecessary nor colliding with the sides of a doorway. Although slightly different critical ratio values have been found in other studies, the use of body size to judge ‘passability’ has been consistently demonstrated [3,4].

This work has been extended into both child and older adult populations. Wilmut & Barnett demonstrated that the critical ratio for passing through an aperture was higher in a group of 8–10 year-olds (~1.6 times shoulder width) than that found in adults (~ 1.45 times shoulder width) [5]. Similarly, in older adults studies have demonstrated that adults aged approximately 68 years show a higher critical ratio of 1.6 than adults aged 23 years who show a critical ratio of 1.4 [6,7]. These group differences lead us to question whether the geometric scaling model fully describes action judgements; if it did, we would not expect any differences in critical ratio once body size had been accounted for (which it had in these studies). Therefore, these results suggest that children and older adults base a decision to turn on more than just body size. Wilmut and Barnett explored what this additional factor could be and found a relationship between the degree of shoulder angle at the aperture and the individual variability in lateral trunk movement as the children approached the aperture [5]. They concluded that body size is important in deciding whether to turn, but that when lateral movement variability is high (as it tends to be in some young children) that this is also taken into account. Similar parameters were considered in the Hackney & Cinelli studies but with no significant relationship found between movement variability and shoulder angle when passing through the aperture [6,7]. However, this could have been due to the small sample size (N = 9) which is not conducive to finding significant relationships. The finding from the Wilmut and Barnett study suggests that children who show a high movement variability and hence low movement consistency will make more conservative judgements. That is they would rotate their shoulders to a greater extent for a given aperture size compared to children with low movement variability. This could be seen as an adaptive strategy which reduces the risk of collision.

Although an appropriate action judgement is important in avoiding collision, of equal importance is the ability to make appropriate changes to movement in order to allow the mover to safely pass. For example, when passing through an aperture it may be appropriate to turn the shoulders. Knowing you need to rotate the shoulders will help to avoid a collision, but only if you are able to adapt your movement appropriately. Studies by Wilmut & Barnett in adults [4] and Wilmut & Barnett [5] in children considered movement adaptations whilst approaching and passing through a range of different aperture sizes. Both the adults and the children approached the aperture in the same way regardless of relative size of the opening, suggesting that a generalised movement is initially generated and later updated to fit the specific demands of the task [4,5]. This fits with the finding that visual information is used only a step or two before an obstacle is reached [8]. Following this initial movement phase, shoulder rotation at the aperture, the size of a reduction in walking speed and the timing of that reduction were all tailored to the exact shoulder to aperture ratio. This finding was consistent across both the adult [4] and the child groups [5]. Once again similar findings have been demonstrated in an older adult population (mean 70 years), with these adults showing significantly larger changes in walking speed at the time of passing the threshold of the aperture for smaller as compared to larger aperture widths [6]. From these results it can be concluded that individuals are able to very precisely determine aspects of the environment, such as aperture size, and to fine tune movement to the exact requirements of the environment.

The research described above shows how typically developing individuals navigate through apertures and how this changes throughout the lifespan, with a demonstration of the importance of movement variability. One population for which movement variability is markedly high is individuals with Developmental Coordination Disorder (DCD). Figures suggest that almost 2% of children in the UK present with DCD [9]; these children display difficulties with fine and gross motor tasks [10] which persist into adulthood, continuing to have a negative impact on everyday life [11]. Anecdotal evidence from children and adults with DCD, their parents and the professionals working with these individuals suggests that they are prone to colliding with obstacles in their pathway [12]. However, there is no clear theoretical basis for this and there is a general paucity of data concerning the locomotor and navigational abilities of individuals with DCD. What there is has predominately focused on very simple walking tasks and the findings are mixed, with some studies reporting marked differences in the walking patterns of children with DCD [13,14] and others reporting few differences in children and adults with DCD [15,16]. Deconinck, Savelsberg, De Clercq, & Lenoir did consider the nature of approaching and stepping over an obstacle in a group of children with DCD [17]. Their results illustrate that although the children with DCD were able to adapt their gait, they did exhibit difficulty controlling momentum due to the increased balance demands that stepping over incurs [17]. To date no studies have considered action judgements in either a stepping or an aperture navigation task in individuals with DCD.

There were two main aims for the current study. The first was to calculate the critical ratio while passing through an aperture in a DCD group compared to a TD group. We would expect a difference here given that degree of rotation at the aperture is linked to movement variability [5] and that individuals with DCD demonstrate higher movement variability than controls while walking [16]. Critical ratio has traditionally been calculated on the basis of the width of the shoulders (as measured by bony landmarks). However, we felt it was also important to incorporate other measures that might impact on the size you perceive yourself to be, your ‘body model’, rather than just shoulder width. These include: the widest part of the upper body (which is not necessarily the shoulders), the left and right lateral movement of the upper trunk while walking and the variability with which we move laterally. We extended previous work by the consideration of each of these factors in addition to the traditional measure of shoulder width. The second main aim of this study was to describe the movement adaptation made while passing through an aperture. Our previous findings have shown that adults approach each aperture in the same way regardless of its relative size, opting to adapt their movement one or two steps prior to crossing the aperture. In the performance of manual tasks research has demonstrated that children with DCD are poor at changing an ongoing movement [18,19] and will adopt strategies to avoid doing this where possible [20,21]. These findings may lead us to expect a difference in the way the adults with DCD approach the aperture depending on whether or not a shoulder turn is needed. Movement adaptations to each aperture size may occur from the very start of the movement, rather than just a step or two before they are needed. Despite a wealth of evidence that children with DCD do not grow out of their movement difficulties [11,22,23] only a handful of studies have considered motor ability in adults with DCD. Since adults have to navigate busy and novel environments more often than children we consider an adults sample in the current study.

## Methods

### Participants

This project was approved by Oxford Brookes University Research Ethics Committee and all participants provided both their written and their verbal consent to take part in this study. This method of seeking consent was approved by the ethics committee. A group of 15 adults with DCD (mean age 25.3 years, range 19.2–34.2 years) and a group of 15 age and gender matched typically developing adults (mean age 25.5years, range 19.9–34.3 years) were included in this study; in each group 6 participants were female and 9 male. Adults with DCD were recruited from two main sources: firstly, from a group known to the authors since having a diagnosis of DCD in their childhood and secondly from a local support group for individuals with coordination difficulties. All participants with DCD were assessed and selected in line with the DSM-5 criteria for DCD and recent guidelines for the assessment of DCD in adulthood (Barnett, Hill, Kirby, & Sugden, 2014). Different assessments were used to ensure that each of the four diagnostic criteria were met. These assessments and the cut-off points employed are described below. For criterion A there is no motor assessment for individuals over 16 years of age that has UK norms, therefore, we carried out both the test component of the Movement Assessment Battery for Children second edition (MABC-2 [25]), which has UK norms for individuals up to 16 years of age) and the Bruininks-Oseretsky Test of Motor Proficiency, Second Edition, Brief Form (BOT-2 Brief [26]), which has US norms for individuals up to 21 years of age). The group of participants with DCD all scored below the 5^th^ percentile on the MABC-2 and below the 18^th^ percentile on the BOT-brief. The Adult Developmental Coordination Disorder Checklist (ADC [27]) and a telephone interview with the participant was used to determine that the motor impairment significantly impacted on activities of daily living (criterion B) and that the onset of these difficulties was during childhood (criterion C). The telephone interview with participants also ensured that the difficulties were not due to a known neurological impairment or intellectual disability (criterion D).

The typically developing (TD) adults were age (to within 6 months) and gender matched to each participant with DCD. These participants completed a telephone interview and the ADC to confirm that no movement difficulties were present. Finally, given the co-occurrence of motor difficulties and attention difficulties all participants (DCD and TD) completed the Conners’ ADHD adult rating scales (CAARS [28]). All participants scored within the average range for the ADHD index indicating no overt attention or impulsivity difficulties.

### Apparatus

**Fig 1:**
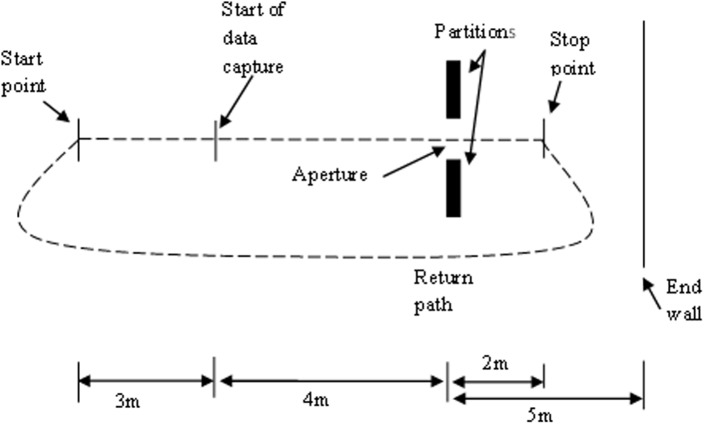
Bird’s eye perspective of the experimental setup. Participants started at the start point 7m away from the partitions. Movement was recorded from 4m away from the aperture up until the stop point (located 2m behind the apertures). Participants then returned to the start point along the return path.

Participants stood 7 m away from the centre of an aperture formed by two sliding partitions (2 m x 1 m). The partitions consisted of a single piece of wood attached to a triangular base supported by castors. When viewed from the front neither the base nor the castors were visible. The back wall of the room lay 5 m behind the partitions. See Fig 1 for an illustration of the setup. A 12 camera Vicon motion capture system (Oxford Metrics) running at 100 Hz was used to track the movement of three 16mm spherical reflective markers placed on the left and right acromion process on the shoulders (LAP and RAP, respectively) and on the seventh cervical vertebrae (C7) at the back of the neck. To determine the point at which a participant passed through the aperture two additional markers were placed on the vertical edge of each partition.

### Procedure

This project was approved by Oxford Brookes University Research Ethics Committee. Initially, shoulder width (the distance between the LAP and RAP) and body width (the widest point on the upper body) of each participant was measured to the nearest mm using digital callipers. The relative sizes of the seven shoulder:aperture (SA) ratios (0.9, 1.1, 1.3, 1.5, 1.7, 1.9 and 2.1) were then calculated for each participant (based on the measurement of the shoulder width not body width). On a given trial, participants were asked to stand behind the start point (7 m from the apertures) and focus on a red circle on the floor in front of their feet (this was so they could not watch the size of the aperture changing or view the experimenter in relation to the aperture). On initiation of a trial participants were instructed to look up and walk, at a self-selected pace, through the aperture to the stop point (2 m past the partitions). Movement was captured from the point at which C7 was 4m from the partitions onwards, thus allowing the participant to have reached a natural walking pace prior to the start of movement capture. On returning to the start point (by passing around the back and to the right of the partitions) participants were told to focus on the circle and not look up until instructed to do so. While the participant returned to the start point, the experimenter changed the aperture size by sliding the partitions closer together or further apart in accordance with a measure placed on the floor. A second experimenter moved the start point by ±30cm in the anterior-posterior direction to prevent the participants from always starting in the same place. Prior to the start of data collection the experimenter demonstrated to each participant what was required. No specific instructions were given on how participants should act when an aperture was too small for them to simply walk through normally; however, when demonstrating the task, the experimenter passed through a narrow aperture by rotating the shoulders. Each aperture ratio was presented 5 times (total of 35 trials per participant) in one of two pseudo-randomised orders, whereby the same aperture was not used on two or more consecutive trials and aperture size did not predictably increase or decrease.

### Data analysis

All participants successfully passed through each aperture size without colliding with either partition. Vicon movement data were filtered using an optimised low pass Woltring filter with a 12 Hz cut-off point and then analysed using tailored MatLab routines. Shoulder width and aperture width were calculated using *x* and *y* positions of the LAP and RAP and the partition markers respectively. Actual aperture width (as determined by the partition markers) was found not to deviate more than ±6.73 mm from desired aperture widths; this error was considered small enough to be negligible. Kinematic measures were taken across two phases of the movement: (1) the approach phase which was defined as the first 2 s of movement capture and (2) the crossing phase, which covered the rest of the movement including passage through the aperture.

In order to measure shoulder angle and movement speed, a number of dependent variables were considered; these are described in Table 1.

**Table 1:** Description of the dependent variables used in this study including the phase during which these were measured.

Variable name	Description of measure	Phase of measurement
***Measurements of shoulder angle***		
Shoulder angle was calculated with respect to the frontal plane at the start point from the *x* and *y* coordinates of LAP and RAP		
Baseline sway (°)	Mean angle rotation of the shoulders across the approach phase	Approach
Shoulder angle at aperture (°)	Angle between the shoulders, with respect to the initial frontal plane, as C7 passed the partitions	Crossing
Variability of shoulder angle (°)	Standard deviation of each participants’ shoulder angle at the aperture for each SA ratio	Crossing
***Measurements of speed***		
For movement speed, the derivative of displacement data was taken and then a least-squares approximation method was used to determine a trend line of a speed-time profile for the movement of C7 during each trial (method as used by Higuchi et al. 2006). This trend line fitted the data well with an R^2^ value of between 0.88 and 0.98. All subsequent measurements of movement speed were taken from this trend line.		
Approach speed (ms^-1^)	Average movement speed during the approach phase (the first 2 s of movement captured)	Approach
Reduction in speed (ms^-1^)	When the change in speed, when a reduction in speed occurred. A reduction in speed occurred if speed after the approach phase, i.e. after the first 2 seconds of movement, dropped more than 3 standard deviations below the approach speed (Higuchi et al. 2006).	Crossing
Time after initiation of reduction in speed (ms)	Movement time remaining after the initiation of the reduction in speed (movement time ended when C7 crossed the aperture). Initiation of reduction in speed was determined as the time of the inflection point prior to speed dropping 3SD below the approach speed (method used in line with the definition of shoulder rotation onset). This variable was only calculated when a reduction in speed occurred.	Crossing
***Measurements of trunk movement***		
Lateral trunk movement (mm)	Average lateral movement of C7 across the approach phase for each trial, then averaged across SA ratio	Approach
Lateral trunk variability (mm)	Standard deviation of the lateral trunk movement across trials for each trial, then averaged across SA ratio	Approach

## Results

### Approach phase

Two-way ANOVAs (SA ratio x group) were used to compare the three approach variables across SA ratio and group (see Table 2 for data). A main effect of SA ratio [F(1,28) = 10.74 p = .003 η^2^ = .28, lower bound report due to violation of sphericity] and an interaction between SA ratio and group [F(6,168) = 4.503 p<.001 η^2^ = .14] was found for approach speed, while no main effect of group was found. Simple main effects demonstrated that this interaction was due to a significant main effect of SA ratio in the DCD group but not in the TD group [DCD: F(6,23) = 11.20 p<.001 η^2^ = .75, TD: p>.05, Pillai’s trace report with Bonferroni adjustment to account for multiple testing]. The effect of SA ratio on approach speed in the DCD group was driven by a slower speed in the 0.9 and 1.1 SA ratio compared to the 1.3, 1.5, 1.7, 1.9 or 2.1 SA ratio (0.9 = 1.1<1.3 = 1.5 = 1.7 = 1.9 = 2.1, Bonferroni adjustment used). In terms of lateral trunk movement a main effect of group was found [F(1,28) = 4.42 p = .045 η^2^ = .14], with the adults with DCD showing a greater lateral trunk movement compared to their matched controls. No main effect of SA ratio nor interaction between SA ratio and group was found for lateral trunk movement. No effect of SA ratio or group or interaction was found for baseline sway.

**Table 2:** Approach phase variables detailed for each SA ratio and each group, standard deviation is given in brackets.

	0.9	1.1	1.3	1.5	1.7	1.9	2.1
Approach speed (ms^-1^)							
TD	1.42 (0.14)	1.42 (0.13)	1.43 (0.15)	1.42 (0.13)	1.43 (0.13)	1.43 (0.13)	1.45 (0.13)
DCD	1.27 (0.16)	1.30 (0.15)	1.33 (0.14)	1.34 (0.14)	1.35 (0.16)	1.35 (0.15)	1.37 (0.15)
Lateral trunk movement (mm)							
TD	42.1 (11.9)	42.1 (11.1)	41.4 (11.4)	40.6 (10.8)	41.0 (11.3)	42.2 (10.8)	40.2 (12.2)
DCD	54.4 (16.1)	52.7 (17.2)	51.6 (17.2)	51.2 (16.2)	51.8 (17.0)	52.1 (16.7)	51.0 (17.7)
Sway (°)							
TD	6.21 (2.45)	5.95 (2.44)	5.75 (2.13)	5.50 (2.05)	5.87 (2.33)	6.05 (2.63)	6.04 (2.48)
DCD	7.02 (2.37)	6.96 (2.43)	6.35 (2.13)	6.67 (2.67)	6.78 (2.31)	6.74 (2.48)	6.60 (2.35)

### Critical ratio

**Fig 2:**
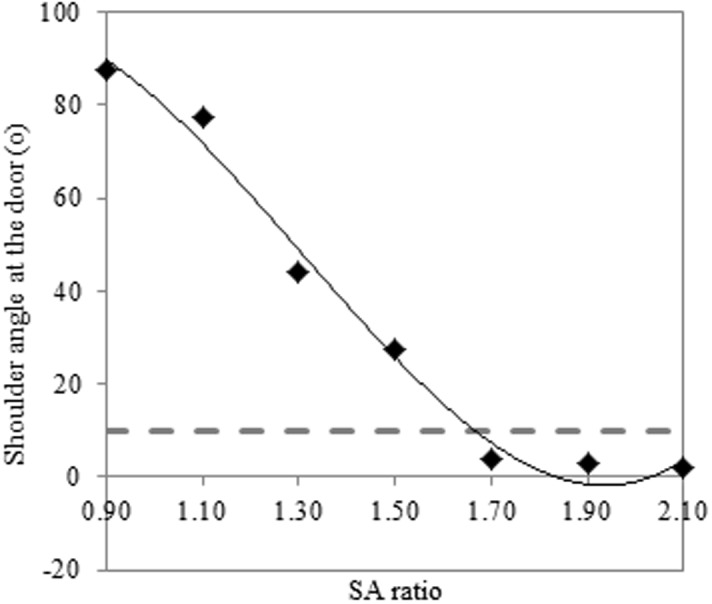
An illustration of the calculation of critical aperture. Data is shown from one participant, blank diamonds illustrate average shoulder angle at the door for each SA ratio and the black line shows the fitted polynomial curve. The dotted line illustrates average baseline sway + one standard deviation of baseline sway. Critical ratio is the point that the two lines intersect.

The critical ratio describes the ratio between shoulder width and aperture width at the point that the participant chooses to rotate the shoulders, i.e. the point at which they no longer perceive that they can pass laterally. This ratio was calculated on the basis of third order polynomial curve fitting to each participant’s profile of shoulder angle at the aperture across the SA ratios. This resulted in an average R^2^ of 0.95 for the TD group and 0.94 for the DCD group (N.B. If nth order polynomial curve fitting was used with the best curve chosen for the given data set (order of fit ranging between third and fifth order), this raised the R^2^ for the typically developing group to 0.98 and the DCD group to 0.96. The result was a reduction in the critical ratio for both groups (1.51 for the TD group and 1.68 for the DCD group). However, the outcome of the statistical analyses remained the same). A critical aperture was then calculated by determining the SA ratio at which the fitted shoulder angle at the door fell at or below one standard deviation above baseline sway (which indicates no overt shoulder rotation). An illustration of this can be found in Fig 2 and the values generated can be found in Table 3. An independent t-test (group) found a significant main effect of group [t(28) = 3.60 p = .001], demonstrating that the SA critical ratio was significantly higher in the DCD group compared to the TD group.

**Table 3:** Critical ratios calculated for shoulder to aperture ratios, body to aperture ratios and body+trunk movement to aperture ratios across the two groups. Standard deviation is given in brackets.

	TD	DCD
Shoulder width (SA ratio)**	1.58 (0.09)	1.75 (0.15)
Body width (BA ratio)*	1.35 (0.09)	1.46 (0.16)
Body width + lateral trunk movement (BTA ratio)	1.24 (0.10)	1.33 (0.18)

* Indicates a group difference at the p<0.05 level.

** indicates a group difference at the P<0.001 level.

In previous work the calculation of the critical ratio has been based only on shoulder width of participants. However, in this study we also measured the widest part of the upper body and found the difference between absolute body width and shoulder width was greater for the adults with DCD (difference 8.2cm) compared to the typical adults (difference 6.8cm). Although there was no significant difference across groups for these values (p>.05) this still could have influenced the critical ratio differently for the two groups. Therefore, we also calculated the critical ratio based on body width (Body to aperture ratio: BA ratio). This calculation changed the values of the critical ratio, however, a main effect of group was still found [t(28) = 2.29 p = .030].

One explanation for the elevated critical ratio in the DCD group could be that both groups judge action capabilities based on anthropomorphic measurements and also on the degree to and consistency with which they move laterally as they approach the aperture (we have shown in the previous section that the DCD group show a higher lateral trunk movement than the TD group). To address this we re-calculated the critical aperture ratio using the sum of body width and the average lateral trunk movement as an indicator of total lateral distance needed to pass through an aperture without turning. This resulted in the new values of critical ratio (Body and trunk variation to aperture ratio: BTA ratio) given in Table 3. An independent t-test demonstrated no group difference in the critical aperture ratio once body width and lateral trunk movement had been accounted for [t(28) = 1.76 p = .090] (If we considered shoulder width and lateral trunk movement and calculated critical ratio with these values we obtain a critical ratio of 1.43 for the TD adults and 1.55 for the adults with DCD. A group difference is seen across these values [t(28) = 2.46 p = .02]).

The lack of the group difference when considering BTA critical ratio suggests that when taking into account body width and lateral trunk movement there is no difference in the action judgements of adults with and without DCD. However, the range of BTA critical ratios for the DCD group was between 1.10 and 1.78, for the typically developing group between 1.06 and 1.42 and so it is clear that at least some of the adults with DCD are continuing to show elevated critical ratios even when body width and lateral trunk movement is taken into account. This could be explained by movement variability which is considered in the next section.

### Movement variability

Any difference in critical ratio between participants may be explained by the consistency at which a participant is able to approach an aperture. Therefore, we considered the lateral trunk movement variability across the groups. For each SA ratio we conducted correlation analyses to see whether this variability measure was related to shoulder angle at the door. Initially shoulder angle at the door rather than critical ratio was used at it allowed us to consider each SA ratio independently rather than either using an amalgamated value. The adults with DCD showed a higher level of lateral trunk movement variability compared to the TD adults as would be expected for this population [F(1,28) = 8.04 p = .008 η^2^ = .22]. For trunk movement variability we found significant correlations for the DCD group for the 1.7, 1.9 and 2.1 SA ratio, with the 1.3 and 1.5 SA ratio approaching significance [1.3: r = .51 p = .053, 1.5: r = .50 p = .058, 1.7: r = .60 p = .017, 1.9: r = .59 p = .022 and 2.1: r = .61 p = .017]. No significant correlations were found between trunk movement variability and shoulder angle at the door for the TD group. Secondly we collapsed the shoulder angle at the door variability across SA ratios and looked for correlations between this and the SA and body width (BA) critical ratio. For the adults with DCD there was a significant relationship for both the SA ratio [r = .58 p = .024] and the BA ratio [r = .55 p = .035]. These relationships show that an individual with greater shoulder angle variability would also demonstrate a higher critical ratio. No significant relationships were seen for the TD adults.

### Crossing phase

#### Movement adaptation: speed

**Fig 3:**
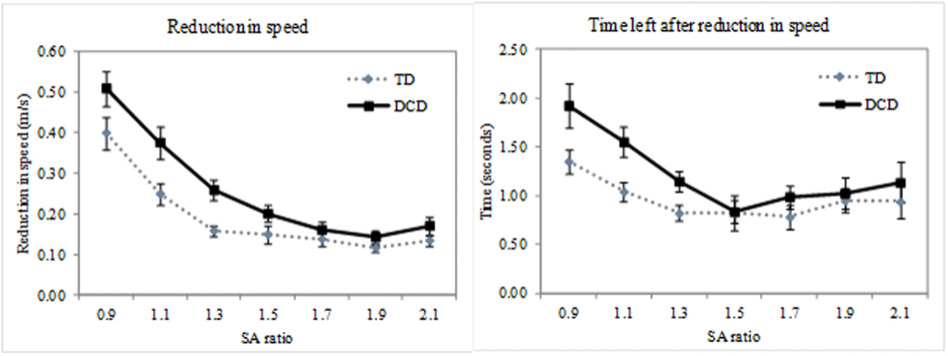
Illustrations of the reduction in speed during the crossing phase across each SA (shoulder-aperture) ratio. Reduction in speed is illustrated on the graph to the left and time left after the reduction in speed is illustrated on the graph to the right. Solid black lines represent adults with DCD (Developmental Coordination Disorder), dotted grey lines represent TD (Typically developing) adults. Error bars represent standard error

A two-way ANOVA (SA ratio x group) found a main effect of SA ratio for both the reduction in speed [F(1,28) = 55.9 p<.001 η^2^ = .67 lower bound reported due to violation of sphericity] and time left after reduction in speed [F(1,28) = 9.84 p<.001 η^2^ = .26 lower bound reported due to violation of sphericity]. In addition, a main effect of group was found for both variables [reduction in speed: F(1,28) = 9.03 p = .006 η^2^ = .24, time left after reduction in speed: F(1,28) = 4.36 p = .046 η^2^ = .14]. Post hoc tests demonstrated that the effect of SA ratio was due to a larger and an earlier reduction in speed for the 0.9 compared to the 1.1 SA ratio and a larger and earlier reduction in speed for the 1.1 compared to the 1.3, 1.5, 1.7, 1.9 and 2.1 SA ratio’s (0.9>1.1>1.3 = 1.5 = 1.7 = 1.9 = 2.1, with Bonferroni correction). The effect of group demonstrates a greater and earlier reduction in speed by the adults with DCD compared to the TD adults. Data for both variables can be found in Fig 3.

#### Movement adaptation: shoulder rotation

**Fig 4:**
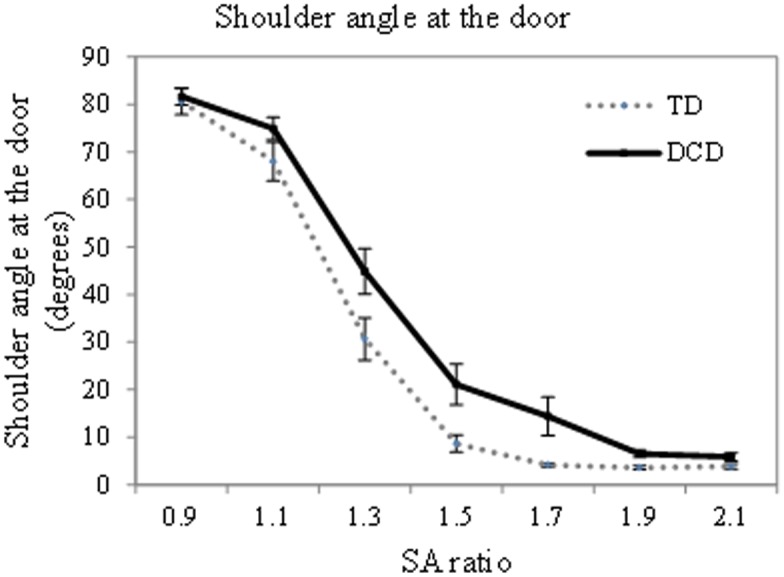
Illustration of the shoulder angle at the aperture. Solid black lines represent adults with DCD, dotted grey lines represent TD adults. Error bars represent standard error

Turn behaviour was investigated by comparing the actual shoulder angle at the aperture; these data can be found in Fig 4. A two-way ANOVA found a main effect of SA ratio [F(1, 28) = 299.34 p<.001 η^2^ = .91 lower bound reported due to violation of sphericity] and a main effect of group [F(1,28) = 11.71 p = .002 η^2^ = .30]. The main effect of SA ratio was due to a greater shoulder angle for the smaller compared to the larger SA ratios (0.9>1.1>1.3>1.5 = 1.7 = 1.9 = 2.1, p<.05 using Bonferroni adjustment). The main effect of group was due to a higher shoulder rotation in the adults with DCD compared to the typically developing adults. In addition a significant interaction was found between SA ratio and group [F(6,168) = 3.00 p = .008 η^2^ = .10]. This significant interaction demonstrates that the pattern of shoulder angle at the door across the SA ratios is different for the adults with DCD as compared to the TD adults. Therefore, the difference between the groups is not simply due to a consistently greater shoulder rotation at each SA ratio. Simple main effects tests were used to further explore this interaction. Once Bonferroni correction had been applied to account for the high number of comparisons a group difference was only found for the 1.9 SA ratio [F(1,28) = 10.98 p = .003]. Therefore when the apertures were at 1.9 times shoulder width the adults with DCD rotated their shoulder significantly more than the TD adults.

## Discussion

This study considered the nature of action judgements and movement adaptations when adults with and without DCD pass through an aperture. We have demonstrated that the ‘critical ratio’ (as measured previously based on shoulder width) is significantly higher in the adults with DCD compared to the typical adults and thus the adults with DCD rotate their shoulders to pass through an aperture for which a TD adult would not turn (even when differences in body size are accounted for). The values for critical ratio quoted in this study are higher than that seen in previous studies. (1.58 compared to 1.3–1.45 in previous studies). This is mainly due to the way in which this value is calculated and there is no gold-standard in the literature. Given that a different method of calculation would result in different final values, but that this would not change the relative difference between the groups (as demonstrated earlier in this manuscript), we suggest that it is not the *value* of critical ratio that is the important factor here, but rather the group differences within a study. In addition to a larger shoulder to aperture critical ratio, the adults with DCD also demonstrated a greater degree of shoulder rotation compared to the typical adults. Unsurprisingly the degree of shoulder rotation decreases as shoulder to aperture ratio increases, however, the pattern of this change is different for the two groups. It is not simply that the adults with DCD always turn an additional ‘n’ degrees, but rather there is a qualitative change in the pattern of turning across the groups. Specifically a group difference is seen at the 1.9 shoulder to aperture ratio with the adults with DCD demonstrating a significantly higher shoulder rotation than the TD adults. From Fig 4 it may seem that a difference should have been found for other SA ratios where the difference between groups was more apparent, however, these other SA ratios demonstrated a higher variance in shoulder angle at the door which may have occluded the difference. It is difficult to draw firm conclusions here as other potential group differences may have been occluded by the statistical correction used to control the Type I error rate.

A higher critical ratio in the adults with DCD reflects the finding that children typically show a higher critical ratio compared to adults [5] and that older adults show a higher critical ratio when compared to young adults [6,7]. These findings suggest that these groups base their action judgements, to turn or not to turn, on more than body size alone. In our previous paper we demonstrated that the degree to which a child rotated their shoulders was influenced by how variable that child’s lateral trunk movement was on the approach and how variable they were in their shoulder rotations. This is an adaptive strategy; if you are a child with a high variability in the amount your trunk moves laterally as you walk and a high variability in the degree to which you rotate your shoulders, then you will not be as consistent in placing your torso in the middle of an aperture or as consistent in achieving a specific shoulder rotation. Therefore, adopting a greater rotation ensures you are small enough (on the frontal plane) to pass through without collision.

In order to better understand the increased critical ratio in the DCD group, we examined whether this could be explained by the increased lateral trunk movement. Once lateral trunk movement was taken into account, the group difference in critical ratio was eliminated. This suggests that when deciding whether or not to turn, participants judge body size based on both body size and lateral body movement, thus ensuring that a collision is avoided. However, when we more closely examined the range of adjusted critical ratios in the DCD group we found that many of the participants with DCD were showing adjusted ratios far higher than those seen in the TD group. This suggests that another factor may also be influencing critical ratio. Previous studies have demonstrated that movement variability is accounted for when judging action capabilities in typically developing children [5] and with work focusing on judgements of action in a stepping paradigm [29]. Given that lateral trunk movement variability was higher for the DCD group compared to the TD group, we went on to consider whether there was a relationship between shoulder angle at the door and lateral trunk movement variability. Our data demonstrate that an adult with a higher lateral trunk movement variability will rotate their shoulders more when passing through an aperture compared to an adult with a low variability. Therefore the elevated critical ratio in some of the adults with DCD could be explained by their lateral trunk movement variability. In sum these results demonstrate that a number of factors play a role in the successful judgement of action capabilities and adults with DCD perform an action which is commensurate with their movement ability to ensure a collision free movement.

In terms of critical ratio, this paper has also demonstrated a marked difference in critical ratio depending on whether body or shoulder width is measured. This alone is unsurprising as body width tends to be wider than shoulder width and this alone changes the critical ratio. What is more important is that when using shoulder width and lateral trunk movement to calculate critical ratio, the adults with DCD show a significantly higher critical ratio than the TD adults. However, when we use body width and lateral trunk movement to calculate critical ratio, that group difference disappears. This finding suggests that researchers must be cautious when choosing how to measure critical ratio, especially when making across group comparisons.

One important consideration at this point is how this finding relates to everyday life. In the introduction we allude to the fact that anecdotal evidence points towards these individuals often colliding with objects. In contrast, in this study we demonstrate that the adults with DCD adopt an adaptive strategy which helps them to avoid this. We do not feel that these points are contradictory. In a lab environment all aspects of the environment are controlled and we typically present a very simple scenario. This is an ideal environment for an adult with DCD to adopt such a strategy; there is ample time, full visual information, and the environment is static. However, in more complex situations (such as the everyday environment) these individuals may be unable to adopt such a strategy and it is under these circumstances that collisions occur. Further research, using more complex environments in the lab is needed to ascertain whether this is the case. Adaptive strategies are seen in various tasks given to participants with DCD, for example, leaving longer traffic gaps before deciding to cross to account for a reduced perceptual ability to detect looming vehicles [30].

One alternative explanation for the elevated critical ratio is that adults with DCD have visuo-spatial difficulties and so make errors in their perception of the size of the aperture (perceiving it to be smaller than it actually is and thus causing the participants to turn more). How accurately adults with DCD perceive aperture size was not the focus of this study and is not currently known, so whether this is an issue remains to be seen. However, poor perceptual abilities in the DCD group would not explain why we see a relationship between lateral trunk movement and shoulder rotation at the door. It is of course possible that visuo-spatial difficulties and motor control difficulties *both* play a part in the elevated critical ratio. Further research is needed to determine whether this is the case.

As in previous papers we considered the task in two parts: the approach phase when no movement adaptation was necessary (as typical adults only adjust a movement a step or two prior to an obstacle) and the crossing phase when movement adaptation was necessary, assuming that a shoulder rotation was needed. In our previous work we have demonstrated that adults and children approach an aperture in the same way regardless of the need to rotate the shoulders [5]. This finding was reflected in the typical adults in the current study, with no difference in approach speed, lateral trunk movement or baseline sway across aperture size. However, the adults with DCD did show a difference in the approach speed, which was tailored to the aperture size. They approached smaller apertures at a slower speed than larger apertures, however, no differences across aperture size were seen in either lateral trunk movement or baseline sway. These findings may point towards a difference in the motor programming of the two groups, with the TD adults using one approach speed regardless of the environment and the adults with DCD using an approach speed seemingly linked to the aperture size. To some extent this fits with the idea that these individuals find adapting an ongoing movement difficult [19–21]. Therefore, the adults with DCD initiate a slower movement for a smaller aperture so that the adjustment is easier later on. This has the added benefit of leaving more time when needing to rotate the shoulders for a very narrow aperture. Following the approach phase both groups generally showed a reduction in speed (although this was not always true for the larger aperture sizes) and this reduction was tailored to the aperture size, with participants slowing more for a smaller aperture and less for a larger aperture. In both groups this reduction in speed was earlier for the smaller apertures and later for the larger; and the timing and degree of slowing was greater and earlier in the movement for the DCD group. Therefore, all groups slow more and slow earlier for smaller compared to larger shoulder to aperture ratios. This finding in typical adults reflects that seen before [5]. One point to note is that the approach phase was defined as the first 2 seconds of movement capture and that it is possible that this incorporated some deceleration. Therefore, the finding that approach speed was variable for different SA ratios for the adults with DCD may be due to these participants slowing earlier for narrower apertures. When considering the speed profiles across the whole trial this did not appear to be the case. However, this alternative explanation cannot be ruled out as a cut-off point is needed to define approach speed. If, despite the speed profiles suggesting to the contrary, the approach speed did include a period of deceleration it still demonstrates that the adults with DCD are approaching the different SA ratios differently and in turn that this is different from the TD adults.

In conclusion, in this study we have demonstrated that adults with DCD show a higher critical ratio compared to TD adults. We conclude that this may reflect that these adults are using an adaptive strategy for passing through apertures, whereby, they include a measure of motor control (lateral trunk movement and lateral trunk movement variability) when perceiving body size and therefore, when making action judgements regarding the decision to turn or not to turn. This adaptive strategy, which results in them turning more often and to a greater degree than their typical counterparts, is coupled with a more cautious approach strategy giving them more time in which to make the necessary movement adaptations to cross the aperture. This resulted in no collisions with the partitions in this study, but we propose that when these individuals are in more complex environments this adaptive strategy may prove impossible to implement, when for example there is not additional time to allow for a slower movement and thus a collision may occur.
